# On the External Application of Belladonna in Delirium Tremens

**Published:** 1854-11

**Authors:** 

**Affiliations:** Physician to the Dumfries and Galloway Infirmary


### SELECTIONS.

*On the External Application of Belladonna in Delirium Tremens.* By Dr. GRIEVE, Physician to the Dumfries and Galloway Infirmary.

Reflecting upon the contracted state of the pupil in the second or developed stage of delirium tremens, Dr. Blake imagined that "by dilating the pupil we might so influence the disturbed visual sense as to dispel, or at least modify, those 'false creations proceeding from a heat-oppressed brain' which characterize this disease, and thus conduce to the comfort and tranquillity of the patient;" and one case is related in which this plan was adopted with apparent benefit. In support of his idea, Dr. Grieve reminds us that the late Dr. Graves proposed the use of belladonna in such cases of fever as were attended with cerebral disease, and contraction of the pupil.

CASE.—On the 25th of March last I was called to attend D.W., aged 49, a man naturally of a robust constitution, but who, of late years, had been much given to intemperance. On inquiry I found that he had been more or less intoxicated for the last three weeks, that he had slept none for several nights in succession, and that the present was his fourth attack of delirium tremens. I found him suffering under great nervous excitement and commotion; laboring under all sorts of optical delusions; fancying that lizards, centipedes, and other entomological horrors were crawling in and around his bed, from which he was convulsively making vain efforts to dislodge them. His pulse was upwards of 120, soft and compressible; his whole body was bedewed with a cold clammy perspiration, and the pupils of both eyes were much contracted. Having obtained some extract of belladonna, I rubbed a little on the eyelids, and remained by his bedside to mark the result. My expectations were soon more than realized, for no sooner was the physiological effect of the drug manifested in the dilated state of the pupils, than the spectral illusions gradually became less and less distinct, the nervous tremors and excitement began to subside, and he soon became comparatively quiescent and tranquil. Soon after this I had the satisfaction to see him fall into the much coveted sleep. Thus I left him; and on revisiting him in a few hours I found that he had slept for two hours; his pupils were then still much dilated; his pulse was below 100, firmer, fuller, and of better character; and altogether his condition, mental and corporeal, was much ameliorated. On interrogating him about his recent hallucinations, he replied, "They were all stuff and nonsense; I see no more of them."

